# Three Days of Measurement Provide Reliable Estimates of Daily Tremor Characteristics: A Pilot Study in Organic and Functional Tremor Patients

**DOI:** 10.5334/tohm.603

**Published:** 2021-04-21

**Authors:** Zeus T. Dominguez-Vega, Gerrit Kramer, Jan Willem J. Elting, Marina A. J. Tijssen, Natasha M. Maurits

**Affiliations:** 1 University of Groningen, University Medical Center Groningen, Department of Neurology, Groningen, The Netherlands

**Keywords:** tremor quantification, long-term accelerometry recording, reliability analysis, organic tremor, functional tremor

## Abstract

**Background:**

Long-term tremor recording is particularly useful for the assessment of overall severity and therapeutic interventions in tremor patients. The purpose of this paper is to investigate the optimal number of days needed to obtain reliable estimates of tremor percentage, tremor frequency variability and tremor intensity in tremor patients using long-term tremor recordings.

**Methods:**

Participants were 18 years or older and were diagnosed with tremor by a movement disorders specialist. Participants wore an accelerometer on the wrist of the most affected arm during 30 consecutive days. Tremor presence, frequency variability and intensity were calculated per day. We used reliability analysis to determine the minimum number of days needed to obtain reliable estimates of these tremor characteristics.

**Results:**

Data from 36 adult organic (OrgT) and functional tremor (FT) patients (24 males; mean age 63.9 ± 11.9 years; 15 FT) were analyzed. Using five hours per day, one day of measurement is enough, except for tremor frequency variability in the OrgT group, where three days are needed and for tremor intensity where two days are always needed.

**Discussion:**

Visual analysis suggested that reliability can be increased considerably by using data from three days instead of one day even when using six hours of data per day. Three days with at least three hours of tremor data provide estimates of tremor percentage, frequency variability and intensity with good to excellent reliability, both for organic and functional tremor.

## 1. Introduction

Tremor is the most common neurological movement disorder [1] and is defined as an involuntary, rhythmic and sinusoidal movement of one or more body parts [2]. Tremor incidence and prevalence increase with ageing affecting more than 4% of the population older than 65 years [3]. More than two-thirds of the population with upper limb tremor faces serious difficulties in daily life.

Longer term tremor recordings might be relevant for the assessment of overall severity and therapeutic interventions [4], particularly for evaluating functional tremor, which has been shown to be less stable over time than organic tremor [5]. In this respect, some studies have successfully quantified and differentiated between tremors [6,7] with the use of long-time tremor recordings.

Several researchers have proposed the use of long-term tremor recordings using EMG [6,8,9,10], ACC [7,11,12,13] or gyroscope [14] signals. In those studies, the most common characteristics used for tremor quantification are tremor intensity, frequency and occurrence. For example, in a study by Parees et al. (2012), functional and organic tremor patients wore an actigraph on the wrist of the most tremulous arm constantly for five consecutive days. Although it was concluded that long-term tremor recordings are needed to objectively quantify tremor presence, no information regarding the minimum number of days needed to quantify tremor in these patient groups was presented. Depending on the aim of the study, recording time varies from less than an hour to analyse tremor frequency [6] or discriminate tremor from other movements in PD patients [12], to several hours to assess fluctuations in tremor amplitude [15] or several (up to five) days to test clinical applicability [10], quantify tremor [9] or quantify tremor presence [7]. There is no consensus regarding the minimum recording time needed for reliable estimation of quantified tremor characteristics using long-term tremor recordings.

Although longer recordings may improve reliability, they may also provide a burden to participants, leading to increased non-wear time and thus reduced data quality. Therefore, minimizing the number of monitoring days will likely positively influence wear-time compliance [15]. To our knowledge only one study [9], addressed the number of days needed to obtain reliable estimation of tremor characteristics in patients with tremor. In this study, the authors argued that tremor quantification in organic tremor patients based on electromyography (EMG) is highly reproducible across three days of consecutive measurement for tremor occurrence, intensity and frequency. Implicitly, their study thus indicates that one day of recording should be enough for reliable estimation of these tremor characteristics in patients with tremor. To determine the minimum number of days needed to obtain reliable estimates of tremor characteristics during long-term tremor recordings we will apply the methods used in measuring daily activity with inertial sensors, i.e., statistical techniques such as correlation [16], ANOVA [16, 17], the intraclass correlation coefficient (ICC) [16,17,18,19] and the Spearman-Brown formula [16,20].

The aim of this pilot study is to determine the minimum number of days needed to obtain reliable estimates of quantified tremor characteristics from long-term tremor recordings using accelerometry, with a focus on tremor presence, tremor frequency variability and tremor intensity in a large group of functional and organic tremor patients, with tremor recording for up to 30 days. This information could be useful for clinicians and clinical researchers who aim to perform long-term home monitoring of tremor patients during unconstrained daily activities.

## 2. Materials and Methods

These data were collected as part of a study on the influence of stress on tremor symptoms in patients with functional and organic tremor [21].

### 2.1 Participants

Patients were recruited from the outpatient clinic of the University Medical Center Groningen (UMCG). Inclusion criteria were being 18 years or older, having a confirmed tremor diagnosis by a movement disorders specialist, being on a stable medication regime and the ability to follow instructions on how to use the device. Clinical guidelines were followed to diagnose the patients. Patients with FT were required to meet the Fahn criteria for probable functional movement disorder, while patients with OrgT were required to meet the criteria for that specific tremor type as judged by a movement disorder specialist [21].

The study was conducted according to the principles of the Declaration of Helsinki (2013), with prior approval of the ethics committee of the UMCG. After written informed consent was given, all patients were instructed (either in the outpatient clinic or at home) on how to attach, remove and use one Shimmer3 (Shimmer sensing, Dublin, Ireland) inertial measurement unit (IMU). All patients were instructed to wear the Shimmer3 IMU on the dorsal side of the forearm, close to the wrist, of the most tremulous limb during daily activities and for 30 days. Participants were also asked to recharge the Shimmer3 IMU during the night and to remove the device during activities which involved the use of water (e.g., taking a shower).

### 2.2 Data acquisition

Before data acquisition in each individual patient, the Shimmer3 IMU was programmed using Consensys v1.2.0 (Shimmer sensing, Dublin, Ireland) with LogAndStream_Shimmer3 v0.8.0 for Bluetooth communication. Subsequently, the Shimmer3 was calibrated using Shimmer 9DoF Calibration v2.3. Calibration prevents misalignment of the electronic board containing the inertial sensors with the outer case and ensures proper recording of sensors. Once calibrated, the IMU was reprogrammed with SDLog_Shimmer3 v0.13 to log data onto the embedded SD card (16 GB) and finally the IMU was configured to use the tri-axial ± 4G (1G = 9.81 m/s^2^) accelerometer and the sampling rate was set to 51.2 Hz. The device was attached to the wrist of the patient using a strap.

### 2.3 Signal processing

All data processing was performed in Matlab (version R2013, Mathworks, Natick, Massachusetts, USA). Our approach is based on a previous study from our group on tremor identification [22]. Data from the 3-axis accelerometer in the Shimmer3 IMU were collected in comma separated values (CSV) files for every day and patient. For the entire analysis, we used the information from the z-axis accelerometry signal. The z-axis signal captures most of the tremor across patients, since the z-axis is perpendicular to the dorsal side of the wrist, and most tremors reflect a wrist flexion-extension movement.

First, wear and non-wear time was determined by analytical assessment and visual verification for each day and patient. We developed an algorithm to detect wear and non-wear time that employed the amplitude of the signal. We hypothesized that segments with an amplitude lower than a certain threshold should be considered as non-wear time segments, similar to van Hees et al. (2011), who used the standard deviation or the value range of the acceleration signal to identify the wear and non-wear time. We first employed noise reduction before threshold identification by using a decimation technique followed by calculating the first derivative of the signal to increase the difference between consecutive points [24]. Hypothesizing that there is low probability for a person not to move at all during three consecutive minutes in a 10 minutes window, we chose to determine wear and non-wear time per ten minutes and thus subsequently segmented the signal into ten minute segments and further into one minute segments. After visual inspection of all signals, we set a value range threshold of 0.4 m/s^2^ (i.e., slightly stricter than the 50 mg = 0.4905 m/s^2^ value range used by van Hees et al., [23]), to identify non-wear segments. Only if at least three consecutive one minute segments were identified as non-wear time, the whole ten minute segment was identified as non-wear time. The threshold was defined by prior visual assessment of the entire data set and results of the entire procedure were then validated by visual inspection of all files. Finally, non-wear time segments were excluded of the tremor identification analysis and remaining wear time segments were concatenated.

To maximize the number of recording days per patient and recording time per day, while keeping as many patients as possible in the analysis, we first visually inspected recording time lengths for all days and patients. We then selected those days for each patient for which at least the required number of hours of data were acquired. Finally, we took the number of hours for the time frame considered per day out of those data, treating it as one continuous wear segment. We defined usable data as those data that remained after periods that the patient was actually using the device were identified.

Once data were organized, tremor features (tremor presence, tremor frequency variability and tremor intensity) were calculated for all usable data. First, to suppress movement artefact, a high-pass zero-phase FIR filter (4th order Butterworth, cut-off frequency = 0.25 Hz) was applied. Subsequently, an existing tremor identification algorithm developed in our group [22] was used to determine the presence of tremor. We briefly repeat the main steps in the algorithm here. First, the filtered acceleration data were further segmented into four seconds windows (without overlapping). Subsequently, power spectral density estimation was performed using the modified periodogram and the presence of a global peak between 3 to 8 Hz was determined (see [22] for details). If such a peak was indeed found and its amplitude was higher than that of global peaks outside the 3–8 Hz band, the window was labelled as containing tremor and the peak frequency was determined. Tremor presence s_{i,k}^{TP} was expressed in the percentage of four second tremor windows in relation to all windows for a day, tremor frequency variability s_{i,k}^{TF} was expressed in the interquartile range of the tremor frequency across all tremor windows for that day and finally, tremor intensity s_{i,k}^{TI} was estimated based on the mean absolute value (MAV) [25] using the acceleration data *x* as follows:

1 MAV = \frac{1}{M}\mathop \sum \limits_{l = 1}^M \left| {x\left(l \right)} \right|

Here, *l* runs over the *M* samples in the time sequence. These tremor characteristics were stored in separate variables for each day *i* (*i* = 1, …, *N_k_*), and patient *k* (*k* = 1, …, 36), where *N_k_* is the number of usable days for patient *k*. This information was sorted in matrices *TP* for tremor percentage, *TF* for tremor frequency variability and *TI* for tremor intensity, where each row corresponded to a patient and each column to a day. If results were not available for a certain day for a certain patient, the matrix element was set to NaN.

### 2.4 Statistical analysis

We analysed the data stored in matrices *TP, TF* and *TI*, per condition (OT or FT), as well as for the whole group. First, to determine whether the tremor characteristic estimates stabilize after a couple of days so that we can determine a ground truth, we plotted the cumulative median (mean subtracted) tremor characteristics over time (days) for each patient. These plots show how and when the estimates for individual patients stabilize when data from more days is taken into account. Using this information, we defined our ground truth as the maximum number of days we have showing a stable value. Second, we verified that data were normally distributed across patients per day, by applying Shapiro-Wilk tests and visual inspection of histograms and QQ-plots. In case of non-normal distributions Log10 transformation was applied to achieve normal distributions. Our statistical approach to determine the minimum number of days needed to obtain reliably estimate the tremor characteristics tremor presence, tremor frequency variability and tremor intensity was adapted from the approach used by Dillon et al., (2016) who determined the minimum number of days needed to estimate habitual activity using long-term measurements with wrist-worn accelerometers. We also took into account the work by Spieker et al., (1995), who investigated the reliability of EMG recordings for tremor quantification across three days. First, we determined Spearman pairwise correlations between tremor characteristic data from any pair of days, to determine the similarity between daily estimates. Here, false discovery rate (fdr) correction was used to correct for multiple comparisons. Next, we used repeated measures analysis of variance (rmANOVA) to determine whether mean tremor characteristics differed across 12 days. In the case of violation of the assumption of sphericity, the Greenhouse-Geisser adjusted F was interpreted. In case of a significant overall F level, posthoc Tukey HSD tests were used to determine which daily estimates differed from each other. Based on Koo and Li (2016), we then used a two-way random effects model with absolute agreement for multiple measurements to calculate ICCs, which compare the proportion of variance in tremor characteristics between patients to total variance (i.e., variance within and between patients). We chose the 2-way random-effects model since we plan to generalize our reliability results to any days, absolute agreement was selected as ICC definition because this concerns that different days assign the same score to the same subject and finally absolute agreement should always be chosen for test-retest reliability studies such as ours because measurements would be meaningless if there is no agreement between repeated measurements (28). An ICC between 0.75 and 0.9 indicates that most of the variance is between patients and not within patients, indicating good reliability of within-patient measurements across days [26]. Only for those cases where the ICC was below 0.75, meaning that one day of measurement is not reliable enough [20], the Spearman-Brown formula was applied to determine how many (additional) days of measurement are needed to obtain a reliable estimate. The Spearman-Brown formula is given by:

2 \frac{{nr}}{{1 + \left({n - 1} \right)r}}

where n is the number of days and r the reliability based on the ICC, in our case. To determine to what extent additional days of measurements might improve the stability of the estimate, we used the cumulative median (mean subtracted) tremor characteristics over time for each patient, again. These plots show how and when the estimates for individual patients stabilize when data from more days is taken into account. We then took the number of days needed to obtain a stable estimate based on this visual analysis and used the Spearman-Brown formula based on the ICCs to assess the resulting increase in reliability. Finally, we also investigated to what extent the number of hours (from 1 to 10) used per day influences the ICC results. Only for those cases where the calculated ICC value was lower than 0.75, we used the Spearman-Brown formula again to determine the number of days needed to obtain an ICC value higher than 0.75 for each case. To execute our analyses, we used R (version 3.5.3: R Core Team, 2019). An alpha of 0.05 was adopted as significance level.

## 3. Results

**Figure 1:**
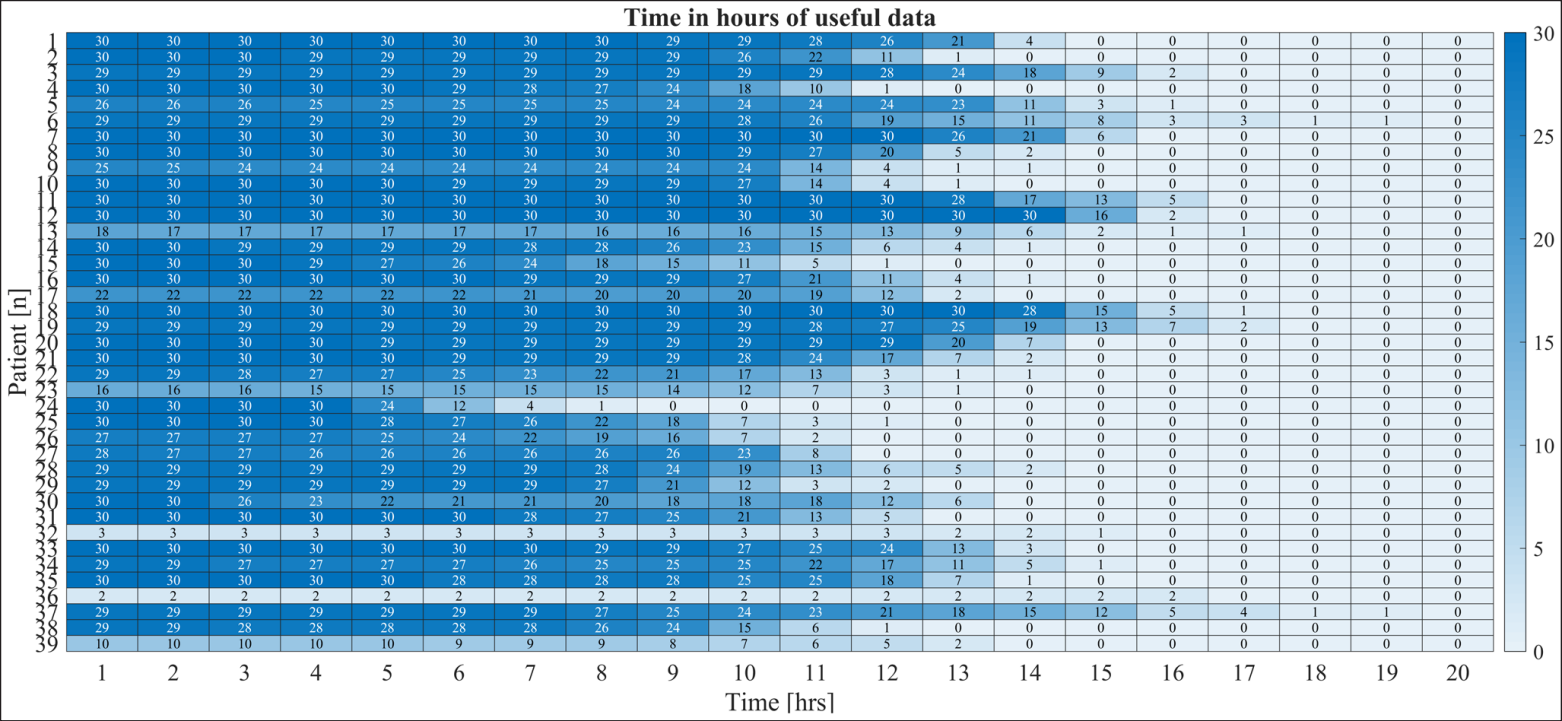
**Number of days with usable data per patient**. Each row represents a patient and each column the number of usable hours from one to twenty. The numbers inside each cell, represent the usable number of days for that patient and number of hours.

Forty-four patients with different forms of at least hand tremor were included of which 39 completed the study; 16 patients with functional tremor and 23 patients with organic tremor. Together, these patients had 1046 days of recorded tremor data. The identification of wear and non-wear time periods resulted in variations in the number of hours per day and days per patient with usable data (see ***Figure 1***) and was visually verified.

Based on visual assessment of ***Figure 1***, we determined that a minimum of 6 hours of usable data per day would be optimal, as it resulted in at least 12 days with usable data for each patient (mean: 26 days, sd: 4 days, range: 12–30). Using this criterion, three patients had to be excluded, leaving 15 patients with functional tremor and 21 patients with organic tremor (6 Parkinsonian, 7 essential, 2 Holmes, 1 enhanced physiological, 2 dystonic and 2 medication induced) for further statistical analysis. For tremor percentage and tremor frequency variability the data from 36 patients were analysed, whereas for tremor intensity the data from one FT patient was removed, this because the information was not properly calibrated for acceleration amplitude, this do not represent an issue for TP or TF estimations.

For these remaining patients, characteristics are given in ***Table 1***.

**Table 1:** **General characteristics of the patients**. Patient characteristics of the functional and organic tremor groups for analyzed patients. SD: standard deviation, IQR: interquartile range.


	FUNCTIONAL TREMOR	ORGANIC TREMOR

Number of patients	15	21

Age (years), mean (SD)	61.5 (12.9)	65.6 (11.1)

Sex (male/female)	9/6	15/6

Disease duration (years), median (IQR)	3.00 (5.25)	10.00 (36.00)

Tremor presence across usable days (%), min-max	6.9–47.3	3.7–85.8

Tremor frequency variability across usable days (IQR in Hz), min-max	1.2–3.8	0.4–2.8

Tremor intensity across usable days (MAV), min-max	0.2–21.2	0.1–15.3


**Figure 2:**
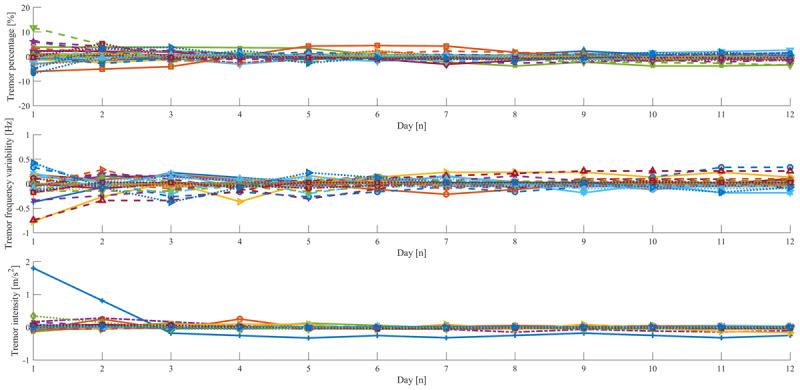
**Cumulative median of the three tremor characteristics**. Mean subtracted plots for tremor percentage (upper) and tremor frequency variability (middle) and tremor intensity (lower). Each line represents a patient.

Plots of the cumulative median tremor characteristics over time for each patient using six hours of data show how and when the estimates for individual patients stabilize, when data from more days are taken into account and allow to decide whether additional days of measurement would add to reliability of the estimates as well as establishing a ground truth for our statistics. To simplify this assessment, we subtracted the mean of the cumulative medians over time before plotting (***Figure 2***). We can observe that after the maximum number of days available (i.e., 12) all estimates have completely stabilized showing that the estimates based on 12 days of data can serve as our ground truth.

Shapiro-Wilk tests and visual inspection of distributions and QQ-plots showed that the data were generally not normally distributed for tremor percentage and tremor intensity, while data were generally normally distributed for tremor frequency variability. After Log10 transformation, tremor percentage and tremor intensity data also became normally distributed. In subsequent analyses, where normally distributed data are assumed (rmANOVA, ICC), we used transformed tremor percentage and tremor intensity data. In ***Table 2***, we present an overview of raw data for all tremor characteristics.

**Table 2:** **Results of the three tremor characteristics**. Summary of the results obtained for tremor percentage, tremor frequency variability and tremor intensity for the three groups (whole group, FT: functional tremor and OT: organic tremor). Results are reported as median (iqr), for non-normally distributed tremor percentage and tremor intensity data and as mean (SD) for normally distributed tremor frequency variability data.


	TREMOR PERCENTAGE				TREMOR FREQUENCY VARIABILITY				TREMOR INTENSITY		
		
	WHOLE GROUP	OT	FT		WHOLE GROUP	OT	FT		WHOLE GROUP	OT	FT

Day1	22.8 (19.1)	24.5 (18.3)	18.6 (18.8)		1.6 (0.6)	1.4 (0.5)	1.9 (0.6)		0.9 (2.6)	0.9 (2.7)	0.9 (2.6)

Day2	23.7 (18.6)	32.5 (18.7)	19.7 (16.7)		1.7 (0.6)	1.4 (0.5)	2.1 (0.5)		1.2 (2.3)	1.3 (2.8)	1.2 (2.1)

Day3	24.0 (15.9)	24.6 (17.6)	17.5 (13.7)		1.7 (0.5)	1.4 (0.4)	2.1 (0.3)		0.9 (2.1)	0.9 (2.1)	1.6 (2.8)

Day4	25.1 (19.3)	29.6 (22.4)	22.5 (10.8)		1.6 (0.6)	1.3 (0.5)	2.0 (0.5)		0.7 (1.1)	0.9 (1.5)	0.6 (0.9)

Day5	23.9 (22.5)	25.1 (22.2)	23.4 (13.9)		1.7 (0.6)	1.5 (0.5)	2.0 (0.6)		0.6 (2.0)	0.6 (3.3)	0.6 (0.7)

Day6	23.9 (21.1)	28.3 (24.0)	17.8 (11.7)		1.8 (0.7)	1.6 (0.7)	2.2 (0.7)		0.7 (1.7)	1.1 (3.5)	0.6 (0.6)

Day7	22.8 (16.7)	27.7 (17.5)	20.3 (11.1)		1.7 (0.6)	1.4 (0.5)	2.1 (0.5)		1.2 (2.4)	1.0 (2.4)	1.4 (2.3)

Day8	21.6 (15.4)	28.4 (22.3)	18.5 (11.4)		1.7 (0.6)	1.3 (0.4)	2.1 (0.6)		0.8 (2.7)	0.6 (1.9)	1.4 (3.0)

Day9	22.3 (19.8)	24.9 (22.2)	15.2 (13.8)		1.7 (0.7)	1.3 (0.5)	2.1 (0.6)		0.7 (2.5)	0.7 (2.6)	0.7 (2.1)

Day10	25.4 (18.6)	27.5 (19.8)	18.6 (12.9)		1.7 (0.6)	1.5 (0.6)	2.1 (0.5)		0.7 (1.9)	0.7 (1.9)	0.7 (1.9)

Day11	23.9 (17.4)	25.6 (16.2)	16.9 (12.4)		1.7 (0.7)	1.4 (0.5)	2.2 (0.6)		0.7 (1.9)	0.9 (1.8)	0.7 (1.8)

Day12	24.3 (17.4)	26.6 (18.3)	18.3 (14.2)		1.7 (0.6)	1.5 (0.5)	2.1 (0.7)		0.7 (1.5)	0.5 (0.9)	0.9 (2.6)


Spearman pairwise correlations between data for each pair of days were significant (all p < 0.05) for all tremor characteristics in most of the cases, indicating linear associations between data for all 12 days. Only for tremor intensity in the FT group, 29 out of 66 comparisons were non-significant. For tremor percentage, the range of pairwise correlations varied across pairs of days within the 12 days and the group. For the whole group it varied between 0.66–0.92, for OrgT between 0.67–0.94 and for FT between 0.47–0.94. The mean pairwise correlations across all pairs of days were 0.84, 0.86 and 0.78 for the whole group, OrgT and FT, respectively. For tremor frequency variability, the range of pairwise correlations varied across pairs of days within the 12 days and the group. For the whole group it varied between 0.58–0.88, for OrgT between 0.33–0.88 and for FT between 0.58–0.94. The mean pairwise correlations across pairs of days were 0.74, 0.65 and 0.78 for the whole group, OrgT and FT, respectively. For tremor intensity, the range of pairwise correlations varied across pairs of days within the 12 days and the group. For the whole group it varied between 0.49–0.81, for OrgT between 0.47–0.89 and for FT between 0.57–0.81. The mean pairwise correlations across pairs of days were 0.65, 0.72 and 0.69 for the whole group, OrgT and FT, respectively.

The rmANOVA results indicated that there were no significant differences between days for means of tremor characteristics, for the whole group, nor for the OrgT and FT groups (tremor percentage: F(11, 385) = 1.165, p = 0.31; F(11, 220) = 1.435, p = 0.158; F(11, 154) = 1.453, p = 0.154 (whole group, OrgT, FT); tremor frequency variability: F(11, 385) = 1.582, p = 0.102; F(11, 220) = 1.091, p = 0.372; F(11, 154) = 1.27, p = 0.246 (whole group, OrgT, FT); tremor intensity: F(11, 261.03) = 1.345, p = 0.225; F(11, 123.86) = 0.624, p = 0.715; F(11, 60.06) = 0.975, p = 0.432 (whole group, OrgT, FT).

The ICC results showed that the variance in tremor characteristics between patients compared to total within- and between-patient variance was generally large, indicating good reliability of within-patient measurements across days. The single measures ICC was 0.84, 0.85 and 0.79, for tremor percentage and 0.77, 0.62 and 0.76, for tremor frequency variability, and 0.71, 0.74 and 0.69, for tremor intensity for the whole group, OrgT and FT, respectively.

Since the ICC for tremor frequency variability in the OrgT group (0.62) and the ICC values for tremor intensity (0.71, 0.74, 0.69) indicated insufficient reliability based on one day of measurement, we applied the Spearman-Brown formula (Eq. (1)), to determine the minimum number of days needed for reliable estimation. For two days, we found that the ICC-based Spearman-Brown reliability is 0.77 for tremor frequency variability in the OrgT group, indicating that for this tremor characteristic and group two days are sufficient for reliable estimation. Similarly, using the Spearman-Brown formula applied to tremor intensity we found that two days are needed to obtain reliabilities of 0.83, 0.84, 0.80 for the whole group, OrgT and FT, respectively.

The above results were obtained using 6 hrs per day and 12 days of data. We also investigated to what extent the number of hours used per day influences the ICC results. In ***Table 3***, we present an overview of the ICC values obtained for different numbers of hours (from 1 to 10) of data used per day.

**Table 3:** **ICC values for the three tremor characteristics**. ICC values for all groups and different time frames from 1 to 10 hrs. Cells with (*) indicate an ICC value higher than 0.75.


		TIME PER DAY (HRS)									

		1	2	3	4	5	6	7	8	9	10

Tremor percentage	Combined	0.72	0.78*	0.80*	0.82*	0.83*	0.84*	0.85*	0.86*	0.87*	0.87*

	OT	0.72	0.79*	0.82*	0.84*	0.84*	0.85*	0.86*	0.87*	0.87*	0.88*

	FT	0.66	0.74	0.74	0.74	0.76*	0.78*	0.80*	0.82*	0.84*	0.82*

Tremor frequency	Combined	0.56	0.64	0.72	0.75	0.76*	0.78*	0.81*	0.81*	0.82*	0.83*

	OT	0.42	0.45	0.51	0.59	0.60	0.63	0.66	0.66	0.67	0.71

	FT	0.53	0.63	0.75	0.74	0.77*	0.78*	0.82*	0.82*	0.83*	0.83*

Tremor intensity	Combined	0.52	0.60	0.64	0.69	0.71	0.71	0.70	0.72	0.74	0.77*

	OT	0.46	0.59	0.65	0.72	0.75	0.73	0.70	0.70	0.72	0.72

	FT	0.60	0.62	0.65	0.65	0.67	0.69	0.71	0.74	0.77*	0.83*


***Table 3*** illustrates that according to the ICC using only a few hours per day does not result in generally reliable estimates of tremor characteristics, when taking a cut-off of ICC = 0.75. Only when using five hours or more per day, the results become generally more reliable across tremor characteristics. These results indicate that tremor percentage is generally the most reliable measure, then tremor frequency and tremor intensity is least reliable.

For 1 to 6 hours of tremor registration we could include 15 (Functional) and 21 (Organic) tremor patients. For 7, 8 and 9 hours of tremor registration one OrgT patient could not be included because of insufficient data. For 10 hours of tremor registration four OrgT patients could not be included for the same reason.

For the cases in ***Table 3*** where the ICC value is lower than 0.75, we used the Spearman-Brown formula to determine the number of days needed to obtain an ICC value higher than 0.75 for each case. In ***Table 4***, we present the ICC values with the number of days needed for each case to obtain reliable estimates for each of the tremor characteristics.

**Table 4:** **Results from the reliability analysis using Spearman-Brown formula**. Reliability analysis using the Spearman-Brown formula, each number in the cell represents the new ICC value and the number between brackets is the number of days needed to obtain such reliability value. Here, NA stands for Non-Applicable since the ICC value for a single day is already higher that 0.75 (***Table 3***).


		TIME PER DAY (HRS)									

		1	2	3	4	5	6	7	8	9	10

Tremor percentage	Combined	0.83 (2)	NA	NA	NA	NA	NA	NA	NA	NA	NA

	OT	0.83 (2)	NA	NA	NA	NA	NA	NA	NA	NA	NA

	FT	0.79 (2)	0.85 (2)	0.85 (2)	0.85 (2)	NA	NA	NA	NA	NA	NA

Tremor frequency	Combined	0.79 (3)	0.78 (2)	0.83 (2)	0.85 (2)	NA	NA	NA	NA	NA	NA

	OT	0.78 (5)	0.77 (4)	0.76 (3)	0.81 (3)	0.81 (3)	0.81 (3)	0.79 (2)	0.80 (2)	0.80 (2)	0.83 (2)

	FT	0.77 (3)	0.77 (2)	0.85 (2)	0.85 (2)	NA	NA	NA	NA	NA	NA

Tremor intensity	Combined	0.76 (3)	0.81 (3)	0.78 (2)	0.81 (2)	0.83 (2)	0.83 (2)	0.79 (4)	0.82 (2)	0.84 (2)	NA

	OT	0.77 (4)	0.81 (3)	0.79 (2)	0.83 (2)	0.84 (2)	0.84 (2)	0.80 (3)	0.82 (2)	0.83 (2)	0.83 (2)

	FT	0.76 (2)	0.81 (3)	0.77 (2)	0.78 (2)	0.80 (2)	0.81 (2)	0.78 (5)	0.83 (2)	0.86 (2)	NA


The results in ***Table 4*** illustrate that, as may be expected, with few hours available we need more days of tremor recording, and with more hours of recording the number of days can be reduced. Using five hours per day, one day of measurement is enough, except for tremor frequency variability in the OrgT group, where three days are needed and for tremor intensity where two days are always needed. The results presented in ***Table 3*** and ***Table 4*** suggest that three days with at least three hours per day of recording could be sufficient to obtain reliable estimates of all three tremor characteristics.

***Figure 2*** also illustrates that even though the statistical analyses show that generally one day is enough for reliable estimation of tremor presence and tremor frequency variability, adding two more days of data to the estimate, stabilizes estimates. Calculating the Spearman-Brown formula for three days for these estimates indeed shows that reliabilities based on the ICC increase to 0.94, 0.94 and 0.92, for tremor percentage, to 0.91, 0.83 and 0.90, for frequency variability, and 0.83, 0.84 and 0.81, for tremor intensity for the whole group, OrgT and FT, respectively.

## 4. Discussion

We determined the minimum number of days needed to obtain reliable estimates of tremor characteristics from long-term tremor recordings using accelerometry in a heterogeneous group of 36 patients with OrgT or FT. We determined estimates of tremor presence in terms of tremor percentage, tremor frequency variability and tremor intensity using six hours of wear-time data across 12 days for all participants. Using a comprehensive set of statistical techniques to assess reliability, we found that one day of recording is enough to obtain acceptable to good reliability, except for tremor frequency variability in OrgT patients, and tremor intensity (whole group, OrgT and FT) in which case two days are needed. Using three days of data was found to stabilize estimates of tremor characteristics, resulting in good to excellent reliabilities. When analysing the effect on reliability of the number of available hours per day, we found that with few hours available we need more days of tremor recording, and with more hours of recording the number of days can be reduced, as may be expected. For clinical applications, based on this pilot study, we suggest to collect three days of tremor accelerometry data for at least three hours per day, both for organic and functional tremor, to obtain estimates of tremor percentage, tremor frequency variability and tremor intensity with good to excellent reliability. We expect our results to be useful for both clinical and clinical research applications that use long-term tremor recordings. During clinical assessment and evaluation of tremor patients, results of long-term tremor recordings could be useful for the clinician to take into consideration. For clinical research, long-term ambulatory tremor assessment could be useful in clinical trials to assess overall severity and the effect of therapeutic interventions.

Apparently, variability in the tremor characteristics studied here is rather limited, so that even in this heterogenous group of patients, one day of six hours of accelerometry recording results in moderate to good and three days in good to excellent reliability. These findings are in line with the one previous study that investigated a similar issue, finding high reproducibility of tremor occurrence, intensity and frequency assessment over three days of EMG measurement in essential and Parkinsonian tremor patients [9]. We have extended our study to include other OrgT as well as FT patients, and used accelerometry recordings, which are more suitable for long-term measurements at home than EMG measurements, because the device can much more easily be taken off and put on again by patients. In our selected group of patients most adequately used the device for 30 days. Even though functional tremor has been shown to be less stable over time than organic tremor [5], we found that both patient groups require similar recording durations to obtain reliable estimates of tremor characteristics, indicating that in our FT patients tremor characteristics are rather stable on the time scale of days.

Although there are no accepted methodologies to determine the minimum number of days needed to obtain reliable estimates of tremor characteristics using ambulatory accelerometry data, the comprehensive battery of statistical tests that we applied is common to assess consistency reliability of questionnaires (see e.g., [27]) and is well accepted in the literature that tries to determine the minimum number of days needed to obtain reliable estimates of daily activity using inertial sensors [16,17,18,19]. In those cases where the ICC was lower to 0.75, using the Spearman-Brown formula allowed to determine how additional days of measurement added to reliability of estimates. Furthermore, the Spearman pairwise correlation results added information regarding the relevance of the order of the days; we now know there is no influence regarding the combination of days we use. Three days are sufficient whether they are the first three days of recording or any other combination of three days throughout the 12 days of measurement.

There are a few limitations to this study. First, our results are limited to the tremor characteristics that we studied; it may be that a different minimum number of days is needed to obtain reliable estimates of other tremor characteristics such as tremor symmetry. Second, the established algorithm [22] that we use to detect tremor during daily activities is not perfect; it correctly classifies 70% of all tremor segments and 96% of all non-tremor segments (compared to the gold standard of a clinical assessor). This means that some daily life activities could be wrongly classified as tremor such as combing hair or brushing teeth [11], and that some actual tremor segments may not be recognized. However, we assume that these inaccurate assessments will be randomly spread across days and will therefore not have a large influence on reliability assessments. Third, even though compared to other studies we included a rather large group of tremor patients that encompassed functional tremor patients for the first time, the total number of patients is still rather small and our results should be reproduced in an independent sample. Finally, there is evidence showing that Parkinsonian tremor patients have mostly rotary movements [5], and some studies have suggested the use of gyroscope signals [14] for tremor quantification. Therefore, whether the minimum number of days needed for reliable tremor characteristic estimation is different when gyroscope signals are used should be further investigated, particularly in Parkinsonian tremor patients.

Although we focused on reliability of daily assessments of tremor characteristics using ambulatory accelerometry recordings, our methodology allows to obtain more fine-grained assessments of these characteristics over the day. This could be particularly useful to assess and monitor temporal patterns in tremor characteristics, e.g., due to medication intake or other interventions. The measures that we have assessed here for their reliability could also be used as part of a diagnostic work-up, e.g., to distinguish between OrgT and FT. However, we here provide no direct evidence that longer term tremor recordings are superior to laboratory testing for the purposes of the differential diagnosis of tremor; that should be further investigated.

## 5. Conclusions

In conclusion, although one day with six hours of accelerometry recording is generally sufficient to obtain estimates of tremor presence, tremor frequency variability and tremor intensity with moderate to good reliability, independent of tremor type, three days of tremor recording increases reliability of these tremor characteristics considerably. For clinical practice, we suggest that three days of ambulatory accelerometry recordings with at least three hours of data from the dominant trembling hand, is sufficient to obtain estimates of tremor presence, tremor frequency variability and tremor intensity with good to excellent reliability in variable forms of tremor.
